# Visuomotor learning promotes visually evoked activity in the medial prefrontal cortex

**DOI:** 10.1016/j.celrep.2022.111487

**Published:** 2022-10-18

**Authors:** Andrew J. Peters, Andrada-Maria Marica, Julie M.J. Fabre, Kenneth D. Harris, Matteo Carandini

**Affiliations:** 1 UCL Institute of Ophthalmology, University College London, London, UK; 2 UCL Queen Square Institute of Neurology, University College London, London, UK

**Keywords:** cortex, sensorimotor, learning, visual, mPFC

## Abstract

The medial prefrontal cortex (mPFC) is necessary for executing many learned associations between stimuli and movement. It is unclear, however, how activity in the mPFC evolves across learning, and how this activity correlates with sensory stimuli and the learned movements they evoke. To address these questions, we record cortical activity with widefield calcium imaging while mice learned to associate a visual stimulus with a forelimb movement. After learning, the mPFC shows stimulus-evoked activity both during task performance and during passive viewing, when the stimulus evokes no action. This stimulus-evoked activity closely tracks behavioral performance across training, with both exhibiting a marked increase between days when mice first learn the task, followed by a steady increase with further training. Electrophysiological recordings localized this activity to the secondary motor and anterior cingulate cortex. We conclude that learning a visuomotor task promotes a route for visual information to reach the prefrontal cortex.

## Highlights

• Visuomotor learning promotes visual responses in the dorsomedial prefrontal cortex
• These visual responses are absent before learning and depend on the visual cortex
• They increase stepwise across training days synchronously with learning
• They occur both in and out of the task context, independent of movement

## Graphical abstract

**Figure:**
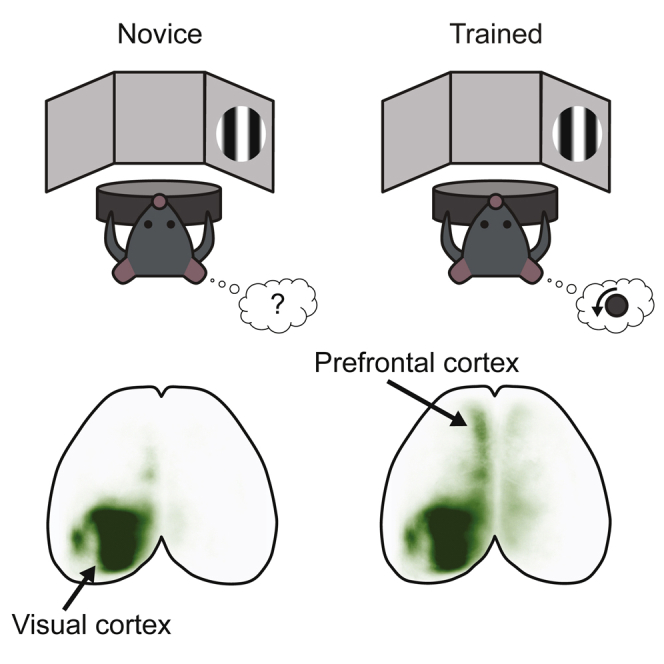

## Teaser

Peters et al. show that visuomotor associative learning promotes visual responses in the mouse dorsomedial prefrontal cortex. These responses are independent of behavioral context. They arise synchronously with learning, with a step-like increase that matches the increase in performance seen on the day when a mouse learns the visuomotor association.

## Introduction

The medial prefrontal cortex (mPFC) is a nexus of sensory processing and motor control (Allen et al., 2017; Harris et al., 2019; Makino et al., 2017) and is causally involved in transforming stimuli into actions (Pinto and Dan, 2015; Siniscalchi et al., 2016; Zatka-Haas et al., 2021). Supporting this role, the mPFC exhibits sensory responses specifically to behaviorally relevant stimuli across modalities (Bichot et al., 1996; Coen et al., 2021; Le Merre et al., 2018; Moorman and Aston-Jones, 2015; Orsolic et al., 2021; Reinert et al., 2021; Wal et al., 2021). However, it is unclear whether these responses are solely sensory or also reflect factors such as attention, learned movement responses, value, or task engagement (Le Merre et al., 2018; Orsolic et al., 2021).

The mPFC is thought to be critical for learning sensorimotor associations (Crochet et al., 2019; Le Merre et al., 2021; Murray et al., 2000). Learning changes its activity (Mulder et al., 2003; Orsolic et al., 2021; Otis et al., 2017; Peters et al., 2021), increasing its sensory responses (Le Merre et al., 2018) and its influence over other cortical areas (Makino et al., 2017). However, it is not known if the increase in sensory-evoked mPFC responses leads, matches, or lags the improvements in behavior that characterize learning.

Moreover, the exact location of mPFC sensory responses is unclear, as the mPFC spans multiple regions, including secondary motor, anterior cingulate, prelimbic, and infralimbic cortex (Le Merre et al., 2021). While midline regions near bregma can respond to visual stimuli even without training (Mohajerani et al., 2013; Murakami et al., 2015; Sreenivasan et al., 2016a), more anterior regions might do so only for trained visual stimuli (Orsolic et al., 2021; Peters et al., 2021; Reinert et al., 2021).

To address these questions, we performed longitudinal wide-field calcium imaging in the mouse dorsal cortex throughout learning of a visuomotor association. Learning promoted selective stimulus responses in the dorsomedial prefrontal cortex (dmPFC) (Le Merre et al., 2021), which increased between sessions alongside behavioral performance. They persisted in passive conditions and after devaluation, indicating that they correlate with the stimuli rather than with learned motor responses, attention, or value.

## Results

**Figure 1:**
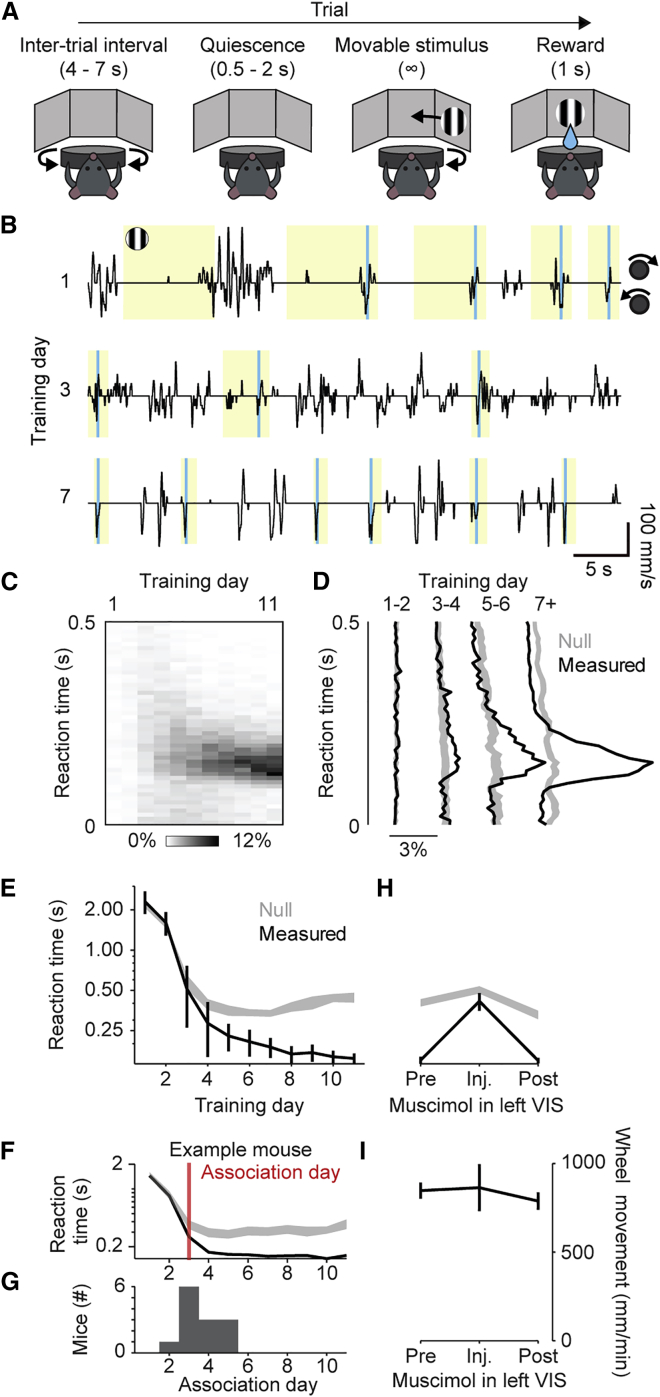
Visuomotor association task (A) Task trial structure, with the timings of each event indicated in parentheses. (B) Example segments of task events and wheel velocity from one mouse across 3 days. The wheel must be turned counterclockwise (downward in this plot) when the stimulus is present (yellow shading) to elicit a reward (cyan lines). (C) Distribution of reaction times as a function of training day, averaged across all mice (n = 13 mice). (D) Distribution of reaction times at different learning points (black) compared with prediction from chance (gray). Curves are mean across mice; shadings, 95% confidence intervals of the null distribution (n = 13 mice). (E) Median reaction times for each training day (black) compared with prediction from chance (gray). Curves and error bars show median ± median absolute deviation across mice (n = 13 mice); shading shows 95% confidence intervals from the null distribution. (F) Median reaction times as in (E) with “association day” for one example mouse. (G) Histogram of association day across mice. (H) Effect of V1 muscimol on reaction times in trained mice, plotted as in (E). Inactivating V1 reversibly increases median reaction times (one-way ANOVA, p = 3.3 × 10^−4^). (I) Effect of muscimol on total wheel movement. Curves and error bars show mean ± SE across mice (n = 5 mice). Wheel movement is not different across groups (one-way ANOVA, p = 0.81).

We trained mice in a visuomotor operant task requiring a single stimulus-movement association. Water-restricted mice were head fixed in the middle of three screens with their forelimbs on a steering wheel. When a grating stimulus appeared on the right-hand screen, turning the wheel counterclockwise moved the stimulus leftward to the center and triggered a sucrose water reward (Figure 1A). Turning the wheel clockwise instead moved the stimulus rightward and triggered a burst of white noise. Stimulus presentations were separated by a random inter-trial interval followed by a random quiescence period, which restarted if the wheel was turned. Therefore, the stimulus appeared at an unpredictable time and only while the mouse was not moving the wheel. This task is derived from a standard two-alternative choice task (Burgess et al., 2017; International Brain Laboratory et al., 2021), but it is simpler because the stimulus was always high-contrast and always appeared on the right side.

Mice learned the visuomotor association in less than 1 week. They learned to move the wheel counterclockwise on the very first day (81% ± 14% of trials, mean ± SD, n = 13), but the timing of these turns was unrelated to visual stimulus onsets (Figure 1B, days 1 and 3). After further training, they began to reliably turn the wheel 100–200 ms after stimulus onset (Figure 1B, day 7), and these turns became more locked to visual stimuli over progressive days (Figure 1C).

To analyze this sharpening of reaction times and distinguish a learned sensorimotor association from random frequent wheel turns, we developed a conditional randomization method. We compared the actual reaction times with a null distribution that was derived from the same wheel turn trajectories but with randomized stimulus onset times obtained by resampling (STAR methods). Under the null hypothesis that actions are unrelated to the visual stimuli, the reaction times would be a random sample from this null distribution and distinguish a learned sensorimotor association from random frequent wheel turns. The decrease in reaction times observed over the first few training days was seen also in the null distribution: it simply reflected an increased propensity to turn the wheel at times unrelated to the visual stimulus (Figure S1A). In later days, however, a peak emerged 100–200 ms post-stimulus, which was not seen in the null distribution, indicating time-locking to the stimulus onset (Figures 1D and 1E). We could thus establish the “association day” for each mouse: the first day when its reaction times diverged from the null distribution (Figure 1F). In most mice, this occurred after 3 days (Figures 1G and S1B).

Even though high-contrast visual stimuli can be processed subcortically (Glickfeld et al., 2013), this association depended critically on the visual cortex. In a subset of trained mice (n = 5), we injected muscimol into the left visual cortex, eliminating visual responses in the left hemisphere (Figure S2A). This inactivation increased reaction times to chance levels (Figure 1H and S2B) but did not affect total wheel movement (Figures 1I and S1A). Thus, mice were equally engaged in the task, but when the visual cortex was inactivated, they were greatly impaired at responding to the visual stimulus.

**Figure 2:**
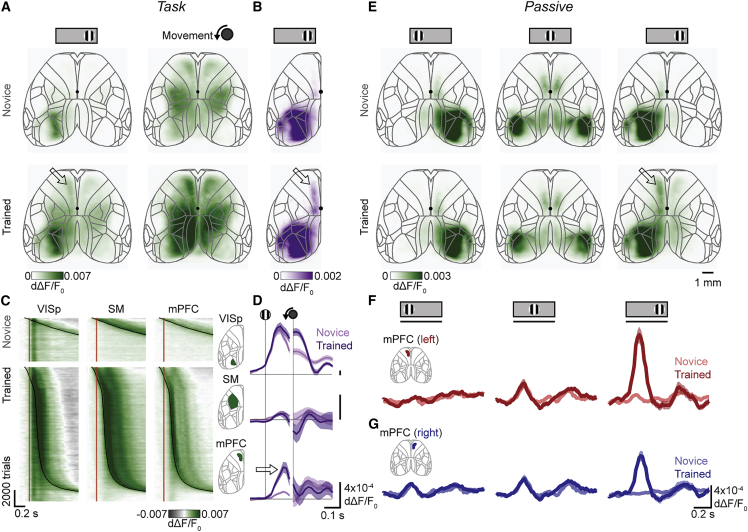
mPFC develops stimulus-evoked responses after learning (A) Mean fluorescence 100 ms after stimulus onset (left) and 0 ms after movement onset (right), averaged across mice (n = 13) in the novice (top) and trained (bottom) learning stage. Values are deconvolved fluorescence relative to baseline (dΔF/F_0_). Arrow: left hemisphere mPFC activity after learning. Outlines are areas from the Allen CCF; black dot is the approximate bregma location. (B) Average stimulus response across mice, computed as the hemispheric asymmetry (h.a.) of the maximum fluorescence 0–200 ms after stimulus onset, to remove bilateral movement-related activity. Arrow: left hemisphere mPFC activity after learning. (C) Fluorescence in left primary visual cortex (VISp), limb somatomotor cortex (SM), and medial prefrontal cortex (mPFC) for all trials and mice, aligned to stimulus onset and sorted by training stage (top, novice; bottom, trained) and reaction time. Red lines, stimulus onset; black curves, movement onset. (D) Hemispheric asymmetry of fluorescence for novice (light purple) or trained (dark purple) mice in the same three regions of interest. Curves and shading show mean ± SE across mice; positive means more activity in the left hemisphere. Arrow: maximum fluorescence 0–200 ms after stimulus onset increases only in the left mPFC after learning (one-way ANOVA, VISp p = 0.33, SM p = 0.72, mPFC p = 2.7 × 10^−4^). (E) Maximum fluorescence 0–200 ms after stimulus onset averaged across mice in the novice (top) and trained (bottom) learning stage, corresponding to the same mice and days as (A) but performed after each task session. Columns are responses to visual stimuli on the left, center, and right. Note the different color scale from (A). (F) Fluorescence during passive stimulus viewing in left hemisphere mPFC in novice (light red) and trained (dark red) learning stage. Curves and shading show mean ± SE across mice. Line under the stimulus icon indicates when the stimulus is on the screen. The left mPFC has an increased response to contralateral (right-hand) stimuli after learning (three-way ANOVA on time, learning stage, and stimulus, learning stage effect p = 2.8 × 10^−26^). (G) As in (F), for the right hemisphere mPFC. The right mPFC has a small response to contralateral (left-hand) stimuli, which does not change with learning (two-way ANOVA on hemisphere and learning stage, hemisphere effect p = 1.5 × 10^−5^, learning stage effect p = 0.37; responses measured as maximum 0–200 ms after stimulus onset). The right mPFC also responds to ipsilateral (right-hand) stimuli but only after learning (three-way ANOVA on time, stage, and stimulus, stage effect p = 3.5 × 10^−19^).

During learning, visually evoked activity emerged in the mPFC. We performed widefield imaging of the entire dorsal cortex on each training day by using mice that expressed GCaMP6s in excitatory neurons (Wekselblatt et al., 2016). After deconvolution, this signal correlates well with the spiking of neurons in deep layers (Peters et al., 2021). To examine how cortical activity changes with learning, we then averaged responses in all days before each mouse’s association day to represent the novice stage and all days after the association day to represent the trained stage. In both stages, we observed activity in the visual cortex after stimulus onset and in the forelimb somatomotor, retrosplenial, and mPFC at movement onset (Figure 2A). However, a key difference was seen in the left mPFC (∼1.0–2.5 mm anterior and 0.5–1.0 mm lateral to bregma), which exhibited increased stimulus-evoked activity in trained mice (Figure 2A, bottom left). The predominantly unilateral nature of this response matched that of visual cortex, suggesting a sensory response to the right-hand visual stimulus.

This visual activity in the left mPFC was followed by motor activity (Figure 2A, right), which acts as a confound because, by definition, trained mice performed more movements in association with the stimulus. To minimize this confound, we exploited the fact that the visual response is predominantly unilateral, whereas movement responses and spontaneous fluctuations are bilateral (Mohajerani et al., 2010, Shimaoka et al., 2019). We thus estimated the hemispheric asymmetry of the response: we subtracted right hemisphere fluorescence from left hemisphere fluorescence after weighting by a constant fit by linear regression during movement with no stimuli (STAR methods). The resulting maps indicated strong asymmetrical stimulus responses in visual cortex, which were expected and present throughout learning, and revealed asymmetric stimulus responses in the mPFC after learning (Figure 2B, arrow).

The increase in visually evoked activity after learning was unique to the mPFC and was not obviously related to task movements. In both the novice and learned stages, the primary visual cortex (VISp) exhibited stimulus-aligned activity (Figure 2C, left), while the somatomotor cortex (SM) exhibited movement-aligned activity (Figure 2C, middle). The mPFC, on the other hand, exhibited only movement-aligned activity before learning but showed additional stimulus-aligned activity after learning (Figure 2C, right). As before, we could isolate stimulus-evoked activity through a weighted hemisphere difference. Stimuli evoked a consistent response in the left visual cortex regardless of learning (Figure 2D, top) and no response in the SM (Figure 2D, middle), while the mPFC gained a new stimulus-evoked response after learning (Figure 2D, bottom, arrow).

Learning a visuomotor association, therefore, appeared to drive the development of stimulus-evoked activity in the mPFC. During the task, however, it is difficult to precisely distinguish stimulus-evoked activity from the overlapping movement-related activity. Therefore, we next analyzed activity during passive viewing, when the mouse viewed identical stimuli but did not perform any action.

The mPFC of trained mice showed visual responses to the trained stimulus even during passive viewing. After each task session, we presented stimuli in a random order on the right screen (identical to the task stimulus), the left screen (not seen during the task), and the center screen (seen in the task during reward delivery and consumption). Turning the wheel had no effect during this passive viewing, and mice—recently satiated from task performance—were often quiescent, holding the wheel still during most stimuli (82% ± 10% of left, 82% ± 12% of center, and 75% ± 16% of right stimulus presentations, mean ± SD across 170 recordings). Nevertheless, to avoid contamination with movement responses, trials with wheel movement were removed from analysis. Activity in the visual cortex (contralateral for left or right stimuli, bilateral for central stimuli) was present throughout training but became more sustained after learning (Figures 2E and S3). A region near bregma in the posterior secondary motor or in the underlying anterior cingulate cortex showed visual responses across training, as expected (Mohajerani et al., 2013; Murakami et al., 2015; Sreenivasan et al., 2016b) (Figure 2E). Consistent with activity during task performance, the left mPFC responded weakly to right-hand (task) stimuli in the novice stage but developed a strong response after learning (Figures 2E–2G, right column). This stimulus also mildly activated the ipsilateral mPFC on the right hemisphere (Figures 2F and 2G, right column). By contrast, the central stimulus (viewed during reward in the task) and the left-hand stimulus (never viewed during the task) elicited no responses in the mPFC on either side (Figures 2E and 2F, left and center columns).

These visual responses in the mPFC depended on the visual cortex because they disappeared when we inactivated the visual cortex with muscimol (Figures S2C–S2E). Thus, learning-induced stimulus responses in the mPFC were not specific to task engagement or satiety and were downstream of responses in the visual cortex.

Visual responses in the mPFC in passive conditions could not be explained by subtle evoked movements. While passive stimulus presentation did not evoke overt limb movements, in trained mice it did evoke subtle behavioral changes such as pupil dilation and whisker twitches (Figures S4A–S4D). These movements varied across trials, allowing us to compare trials with similar amounts of whisker movement in novice and trained stages (Figure S4E). Movements were accompanied by consistently low mPFC fluorescence in novices and high mPFC fluorescence after learning (Figures S4F and S4G). Therefore, the increased visual responses in the mPFC of trained mice could not be accounted for by the behavior we observed on video.

The visual responses in the mPFC of trained mice persisted across long-term devaluation. In some mice (n = 3), we recorded passive responses to visual stimuli for 3 weeks after task sessions were ended and mice had free access to water. The mPFC continued to respond to the trained right-hand visual stimulus, indicating that the visually evoked responses were robust to devaluation: they were not extinguished by loss of motivation or repeated passive exposure with no associated task (Figure S5).

**Figure 3:**
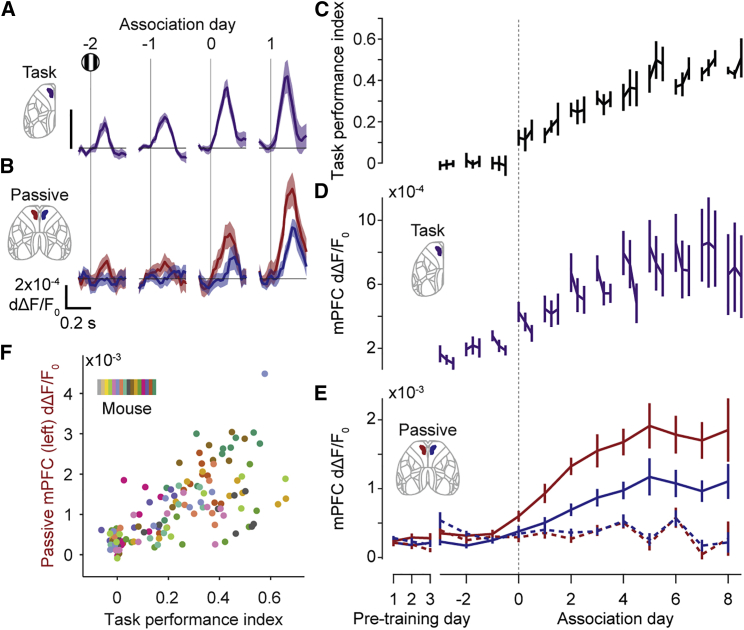
mPFC stimulus-evoked responses track behavioral performance across learning (A) Fluorescence in the mPFC as hemispheric asymmetry during task performance, aligned to stimulus onset and plotted relative to association day. Positive means more fluorescence in the left hemisphere; day 0 is the association day. Curves and shading show mean ± SEM across mice (n = 13 mice). Maximum fluorescence 0–200 ms after stimulus onset increases specifically on the association day (signed-rank test, day −2 versus −1 p = 0.23, day −1 versus 0 p = 6.1 × 10^−3^). (B) Fluorescence in the left (red) and right (blue) mPFC during passive viewing of right-hand stimuli. Curves and shading show mean ± SEM across mice (n = 13 mice). Maximum fluorescence 0–200 ms after stimulus onset increases specifically on the learned day (signed-rank test, day −2 versus −1 p = 0.62, day −1 versus 0 p = 2.4 × 10^−3^). (C) Task performance relative to association day. Performance is quantified as “task performance index,” defined as the difference divided by the sum of actual and chance median reaction times (positive values indicate shorter reaction times than chance). For each day, the three connected points represent equal thirds of trials for each day. Curves and error bars show median ± m.a.d. across mice (n = 13 mice). Performance improves after, rather than within, the day before association (first third versus last third of trials on association day −1, signed-rank test p = 0.45, last third of trials on association day −1 versus first third of trials on association day 0, signed-rank test p = 0.017). (D) Fluorescence in the mPFC as the maximum h.a. (STAR Methods) 0–200 ms after stimulus onset during the task, relative to association day and split into thirds of trials as (C). Curves and error bars show mean ± SEM across mice (n = 13 mice). Activity increases across, rather within, days (activity differences are larger between the first third of trials across days compared with the first and last third of trials within days, signed-rank test p = 4.9 × 10^−4^). (E) Maximum mPFC fluorescence 0–200 ms after stimulus onset during passive viewing, relative to association day and including 3 days prior to training (“pretraining days”). Activity is shown for the left (red) and right (blue) hemisphere mPFC and for right-hand stimuli (solid lines, stimulus used in task) and left-hand stimuli (dotted lines, stimulus not used in task). Curves and error bars show mean ± SEM across mice (n = 13 mice). (F) Relationship between passive sensory responses in the mPFC and task performance across mice and days. The x axis shows the responses of the left mPFC to right-hand stimuli in the passive condition; the y axis shows “task performance index” as in (C). Colors represent mice, and each dot represents one day.

Visually evoked responses in the mPFC closely tracked behavioral performance across days and increased according to the learning rate of each individual mouse. Because different mice learned the task at different rates, we examined the time course of activity changes relative to the association day (when performance first exceeds chance). Stimulus-evoked mPFC activity increased substantially on the association day, both in the task and during passive viewing (Figures 3A and 3B). Remarkably, this increase manifested as a step-like change in both behavior and activity at the beginning of the session rather than a gradual improvement during the session (Figures 3C and 3D). Both performance and mPFC responses then increased with further training to reach a plateau approximately 5 days after the association day, which was mirrored by mPFC responses to stimuli presented passively at the end of each training session (Figures 3C–3E). Furthermore, there was a strong mouse-by-mouse correlation between performance in the task and passive stimulus responses in the mPFC measured on the same day (Figures 3F and S6). These results show that sensory responses evolve in mPFC concomitant with behavioral learning, with both exhibiting an initial jump at the start of the association day, followed by an increase across subsequent training.

**Figure 4:**
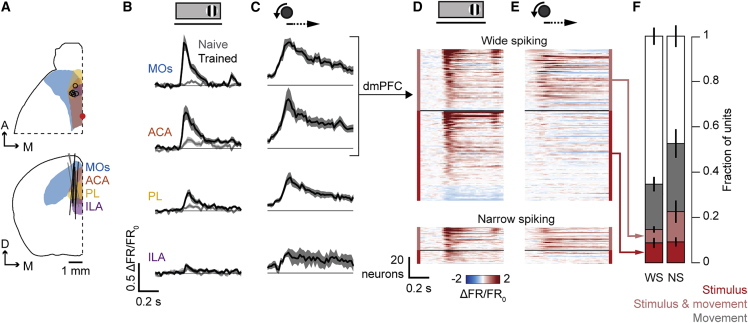
mPFC neurons of trained mice show stimulus- and movement-evoked responses in secondary motor and anterior cingulate cortex (A) Neuropixels probe locations for naïve (gray lines, n = 4 mice) and trained (black lines, n = 5 mice) mice. Area outlines are secondary motor (blue), anterior cingulate (orange), prelimbic (yellow), and infralimbic (purple) cortex. Red dot represents bregma. (B) Multiunit activity aligned to onset of right-hand stimuli during passive viewing in naive (gray) and trained (black) mice for four frontal areas, including secondary motor (MOs), anterior cingulate (ACA), prelimbic (PL), and infralimbic (ILA) cortex. Curves and shading show mean ± SEM across recordings (n = 12 naive, 14 trained). Line under stimulus icon indicates when the stimulus is on the screen. Stimulus responses are larger in trained mice in all areas except ILA (two-way ANOVA, interaction effect MOs p = 6.3 × 10^−31^, ACC 3.9 × 10^−30^, PL p = 1.7 × 10^−8^, ILA p = 0.94). (C) Multiunit activity aligned to onset of spontaneous movements during delay periods of the task (without stimuli), from the same recording days and areas in (B) for trained mice. Curves and shading show mean ± SEM across recordings. Note that naive mice are not included as they do not spontaneously move the wheel. Movement onset time corresponds to left edge of arrow above plots. (D) Pseudocolored peri-stimulus rate histograms of populations of wide spiking (top) and narrow spiking (bottom) neurons in the dmPFC, aligned to stimulus onsets during passive conditions (without movement). Neurons above the black horizontal line have significant responses to stimulus and movement (p < 0.01, shuffle method) and below have significant responses to stimulus only. (Neurons responding to movements only or to neither are not shown.) Within each group, neurons are sorted by stimulus response amplitude. Color indicates change in rate relative to prestimulus baseline rate. (E) Peri-event rate histograms of neurons plotted as in (D) but aligned to movement onset during delay periods of the task (without stimuli). (F) Fraction of wide spiking (WS) and narrow spiking (NS) neurons responding positively or negatively to right-hand stimuli during passive viewing (red; p < 0.01, shuffle method), to movement onset during task delay periods with no stimulus (gray), and to both (pale red) in the dorsomedial prefrontal cortex (dmPFC; defined as MOs and ACA) of trained mice. Values and error bars represent mean and SEM across recordings (n = 10 recordings). Movement-only responses and stimulus-and-movement responses are more prevalent in NS cells, but stimulus-only responses were similarly prevalent between populations (shuffle test of cell type within experiment and area, stimulus only p = 0.44, stimulus and movement p < 0.001, movement only p = 0.01).

The mPFC activity we observed with widefield imaging reflected spiking of neurons located primarily in areas of the dmPFC: the secondary motor and the anterior cingulate cortex. The mPFC comprises two subregions, which each encompass two areas: the dmPFC, which includes the secondary motor (MOs) and anterior cingulate (ACA) cortex, and the ventromedial PFC (vmPFC), which includes the prelimbic (PL) and infralimbic (ILA) cortex (Le Merre et al., 2021). To determine which of these areas within the mPFC contained neurons with stimulus-evoked activity, we performed acute electrophysiological recordings in trained mice (10 recordings across 5 mice, all of whom had previously undergone widefield imaging). We targeted Neuropixels probes to the area exhibiting stimulus responses after learning based on widefield fluorescence, which were histologically verified to pass through all four mPFC regions (MOs, ACA, PL, and ILA) (Figure 4A). Multiunit signals showed visual responses selectively to the trained right-hand stimulus, in a decreasing dorsal-to-ventral gradient, with the largest responses in the dmPFC (Figure 4B, black curves). By contrast, and as expected from imaging, mPFC neurons in naive mice exhibited essentially no stimulus-evoked spiking (12 recordings across 4 mice; Figure 4B, gray curves).

The visual responses of dmPFC were carried by few neurons, which often also responded to movement. Multiunit activity in the dmPFC was evoked not only by stimuli but also by delay-period movements (with no visual stimuli), indicating a mixing of sensory and motor responses (Figure 4C). To determine whether responses to stimuli and movements were present in the same cells, we quantified responsiveness for high-quality single units. Stimulus responses in the dmPFC were relatively rare (16% ± 10% SD, n = 10 recordings), while movement responses were more common (28% ± 12% SD, n = 10 recordings). Stimulus-responsive units, however, were more likely to respond to movement than expected from chance by shuffling movement-responsive classification within experiment (p < 0.001). Consistent with previous reports (Kim et al., 2021; Pinto and Dan, 2015), stimulus responses were more common and more likely to be accompanied by motor responses in narrow spiking neurons (putative fast-spiking inhibitory interneurons) than in wide spiking neurons (putative excitatory neurons) (Figures 4D–4F). These results indicate that the stimulus-evoked response in the mPFC arises from a subset of neurons that commonly also respond to movements.

## Discussion

Our results show that the mPFC becomes responsive to a visual stimulus concurrently with learning a visuomotor association and that both performance and visual responses in the mPFC increase between training sessions but not within sessions. These findings suggest that stimulus information becomes routed to the mPFC through learning, which in turn may drive execution of the associated movement.

Our results support the notion that sensorimotor learning is tied to the propagation of sensory signals into the mPFC, where they may provide context for movement (Barthas and Kwan, 2017; Heilbronner and Hayden, 2016; Rushworth et al., 2011). Indeed, the mPFC is necessary to execute visuomotor associations (Coen et al., 2021; Zatka-Haas et al., 2021), with stimulus-locked activity being particularly critical for performance (Zatka-Haas et al., 2021). This activity is likely to be inherited from the visual cortex, perhaps through direct projections from secondary visual regions (Itokazu et al., 2018; Kim et al., 2021), since it was abolished when we inactivated the visual cortex.

By contrast, our data do not seem to be explainable through mPFC representations of stimulus value, i.e., capacity to predict rewards. Multiple mPFC areas have been associated with stimulus value, including MOs (Sul et al., 2011), ACA (Kennerley et al., 2011), and PL (Lak et al., 2020). However, the mPFC did not respond to central stimuli, even though these stimuli were present whenever reward was delivered. Moreover, the mPFC responded to task stimuli even after long-term stimulus devaluation, when they were unlikely to cause an expectation of reward.

We found that both behavior and mPFC activity change between days rather than within days, suggesting that learning involves plasticity after training. While some types of association can be developed within a session (Komiyama et al., 2010), it is common to observe across-day changes in neural activity (Costa et al., 2004). These changes may result from spine growth and be facilitated by activity replay after each day’s training (Peyrache et al., 2009; Yang et al., 2014). In our task, mice learn to turn the wheel on the first day, but at times unrelated to the visual stimulus, before developing a sensorimotor association between stimulus and movement. These untimed movements occurring early in training are also accompanied by mPFC activity. We hypothesize that after early training, mPFC activity is sufficient to drive movements and that synaptic plasticity occurring between later training sessions routes visual activity to the mPFC, causing the stimulus to trigger the movement.

The mPFC is likely part of a larger network that together drives movement responses to stimuli. We found that sensory and motor responses overlapped in mPFC regions and even within neurons (Pinto and Dan, 2015). These responses may serve multiple functions downstream; for example, sensory mPFC activity may influence perception across modalities including visual (Huda et al., 2020; Zhang et al., 2014), auditory (Schneider et al., 2018), and somatosensory (Manita et al., 2015). In turn, motor mPFC activity may coordinate motor commands within the motor cortex (Allen et al., 2017; Makino et al., 2017), superior colliculus (Huda et al., 2020), and striatum (Otis et al., 2017; Peters et al., 2021).

The exact prefrontal region that developed stimulus responses after learning lies within the dmPFC, which includes the MOs and ACA cortex. This region is medial to those often associated with delay-period activity and higher-order task and movement variables (Svoboda and Li, 2018). It is also distinct from the orofacial anterior-lateral motor (ALM) region (Komiyama et al., 2010), and likely distinct from the rostral forelimb area (RFA) (Tennant et al., 2011), vibrissal motor (vM1) cortex (Ferezou et al., 2007), and medial motor (MM) area (Chen et al., 2017). This region is also anterior to a different medial frontal region that exhibits responses to visual stimuli in novice mice (Mohajerani et al., 2013; Murakami et al., 2015; Sreenivasan et al., 2016a), demonstrating two functionally distinct, visually responsive medial frontal regions. However, it possibly overlaps with or is homologous to the frontal orienting fields (FOFs) found in rats (Erlich et al., 2011). It seems unlikely to be specifically associated with forelimb movements, given a previous demonstration that this region is involved in a licking visuomotor task (Goard et al., 2016). This region of the dmPFC might therefore bear a unique relationship to learning.

### Limitations of the study

Our results show a striking correlation between mPFC sensory responses and behavioral visuomotor association, but they do not demonstrate that mPFC activity is causal toward either the learning or the behavior. We demonstrate that both behavior and mPFC stimulus responses change more across days than within days, but mice become progressively more sated within each day, which may affect both reaction times and mPFC responses to mask within-day learning. We found that visually evoked responses in the mPFC depend on the visual cortex, but we did not assess whether they reflect direct or indirect inputs from the visual cortex. Finally, while we found that stimulus responses in the mPFC were present regardless of small facial movements, this does not rule out a role of other possible small movements that we could not observe.

## STAR★Methods

### Key resources table

**Table:** 

REAGENT or RESOURCE	SOURCE	IDENTIFIER
**Chemicals, peptides, and recombinant proteins**		

Muscimol	Sigma Aldrich	CAS number: 2763-96-4
DiI	ThermoFisher Scientific	Catalog number: V22885

**Deposited data**		

Data to produce figures	https://osf.io/2wh5v/	The Open Science Framework: https://doi.org/10.17605/OSF.IO/2WH5V

**Experimental models: Organisms/strains**		

Mice tetO-GCaMP6s	The Jackson Laboratory	Jax #024742, RRID:IMSR_JAX: 024742
Mice CaMK2a-tTa	The Jackson Laboratory	Jax #007004, RRID:IMSR_JAX: 007004

**Software and algorithms**		

Code to produce figures	https://github.com/petersaj/Peters_et_al_CellReports_2022	Zenodo: https://doi.org/10.5281/zenodo.7043441
MATLAB > R2018a	MathWorks	https://www.mathworks.com
Rigbox	Bhagat et al., 2020	https://github.com/cortex-lab/Rigbox
Open Ephys GUI	Open Ephys	https://open-ephys.org/gui
Kilosort 2.0	Stringer et al., 2019	https://github.com/MouseLand/Kilosort/releases/tag/v2.0
Phy	Rossant et al., 2016	https://github.com/cortex-lab/phy
DeepLabCut	Mathis et al., 2018	https://github.com/DeepLabCut/DeepLabCut
AP_histology	Peters et al., 2021	https://github.com/petersaj/AP_histology
Bombcell	This paper	https://github.com/Julie-Fabre/bombcell

**Other**		

Norland fast-curing optical adhesive	Norland Products	NOA 81

### Resource availability

#### Lead contact

Further information and requests for resources should be directed to and will be fulfilled by the Lead Contact, Andrew Peters (peters.andrew.j@gmail.com).

#### Materials availability

This study did not generate new unique reagents.

### Experimental model and subject details

#### Animals

All experiments were conducted according to the UK Animals (Scientific Procedures) Act 1986 under personal and project licenses issued by the Home Office. Mice were transgenic (tetO-G6s;Camk2a-tTa (Wekselblatt et al., 2016)) male and female adults (6 weeks or older). Mice were group-housed when possible and kept in standard 12-hour light/dark cycles with *ad libitum* access to food. Mice engaged in behavioral training were provided water through water rewards during task performance and supplemented with water afterwards to reach a daily required amount. Mice not engaged in behavioral training were provided water *ad libitum*.

### Method details

#### Surgery

Surgery for widefield imaging involved affixing a headplate and plastic well to the skull and applying an optically transparent adhesive to the skull. Mice were anesthetized with isoflurane, injected subcutaneously with Carprieve, and placed in a stereotaxic apparatus on a heat pad. The head was then shaved, the scalp cleaned with iodine and alcohol, and the scalp and periosteum were removed to expose the skull. The cut skin was sealed with VetBond (World Precision Instruments), and a custom steel headplate was fixed to the interparietal bone with dental cement (Super-Bond C&B). A plastic 3D-printed U-shaped well was then cemented to enclose the edges of the exposed skull. A layer of VetBond was applied to the skull followed by two layers of UV-curing optical adhesive (NOA81, Norland Products). Carprieve was added to the drinking water for 3 days after surgery.

For muscimol injections, the retinotopically peripheral zone of V1 in the left hemisphere (contralateral to the trained stimulus) was targeted using widefield retinotopic mapping relative to vasculature. Mice were anesthetized with isoflurane, injected subcutaneously with Carprieve, and head-fixed using the previously implanted headplate. The optical adhesive was drilled away over the targeted location and a small craniotomy was drilled. Muscimol (Sigma, 5 mM in ACSF) was injected through a sharpened ∼40 μm borosilicate capillary with a pneumatic injector (Nanoject, Drummond Scientific) in two boluses of 70 nL at 300 and 700 μm from the cortical surface. The craniotomy was then filled with Kwik-Sil (World Precision Instruments), a thin layer of clear dental cement was applied, and overlying optical adhesive was replaced. Recordings were performed at least 1.5 hours after muscimol injections.

For electrophysiological recordings, the mPFC in the left hemisphere was targeted using widefield responses to right-hand stimuli relative to vasculature (centered at approximately 1.7 mm AP and 0.7 mm ML relative to bregma, Figure 4A). Mice were anesthetized with isoflurane, injected subcutaneously with Carprieve, and head-fixed using the previously implanted headplate. The optical adhesive was drilled away over the targeted location and a small craniotomy was drilled. The craniotomy was covered with Kwik-Cast (World Precision Instruments), and recordings were performed at least 1.5 hours after surgery.

#### Visuomotor operant task

Mice performed an operant task where a visual stimulus on the right-hand side could be moved with a wheel into the center to receive a water reward. Mice were water restricted and typically received their required water amounts during the task, or were otherwise supplemented to a minimum relative to the body weight of each mouse. The task was a variant of one described previously (Burgess et al., 2017) and programmed in Signals, part of the Rigbox MATLAB package (Bhagat et al., 2020).

Visual stimuli consisted of square gratings with 100% contrast, 1/15 cycles per degree, and randomized phase on each trial, within a circular gaussian window of σ = 20° which effectively covered an entire screen. At the start of each trial, the visual stimulus appeared on the right-hand screen and was positionally yoked to wheel movements, for example with counterclockwise (leftward) movements of the wheel bringing the visual stimulus leftward towards the center. If the stimulus was brought to the center a 6 μL water reward was delivered, and if the stimulus was instead moved rightward off the screen a low burst of white noise was played through speakers under the screens. In a rewarded trial, the stimulus was fixed in place on the center screen for one second while the mouse consumed the water. Mice quickly learned to move the wheel leftward instead of rightward, with 81 ± 14% of rewarded trials on day 1 and 96 ± 3% across-session average (mean ± s.d. across 13 mice).

Between the response of one trial and the appearance of the visual stimulus on the next trial, two delay parameters were independently and randomly selected for each trial. The first, an inter-trial interval (ITI), was a fixed time from the outcome of the previous trial. The second, an enforced quiescence period, was a timer that began after the ITI and would reset with any wheel movement. Delay timings were shorter at the start of training and were lengthened once mice first obtained their full daily water amount in the task, which was usually on the first or second day of training. Delay timings were selected from a range in 100 ms increments, with initial ranges being 1–3 s for ITIs and 0.5–1 s for quiescence periods, which were increased to 4–7 s for ITIs and 0.5–2 s for quiescence periods.

Training days were not always consecutive, and in instances where a day was skipped between training, mice were provided with a minimum amount of water in their home cage.

#### Passive stimulus presentation

Passive stimulus presentation was performed for 3 days before training to serve as acclimation to the recording rig and to provide a baseline response to visual stimuli. Passive stimuli were also presented after each session of task performance. The stimuli presented were gratings of the same size and spatial frequency as those presented during the task, but on either the left screen (never seen during the task), the center screen (seen during reward in the task), or the right screen (seen at the start of each trial during the task). The order of the three stimuli was randomized for each presentation, and all stimuli were presented 50 times. Stimuli were presented for 500 ms, with a 2–3 s inter-stimulus interval randomly chosen in 100 ms increments. All stimulus presentations with wheel movement were excluded from analysis.

#### Widefield imaging and fluorescence processing

Widefield imaging was conducted with a sCMOS camera (PCO Edge 5.5) affixed to a macroscope (Scimedia THT-FLSP) with a 1.0x condenser lens and 0.63x objective lens (Leica). Images were collected with Camware 4 (PCO) and binned in 2x2 blocks giving a spatial resolution of 20.6 μm/pixel at 70 Hz. Illumination was generated using a Cairn OptoLED with alternating colors, yielding a 35 Hz signal for each color. Blue light (470 nm, excitation filter ET470/40x) was used to capture GCaMP calcium-dependent fluorescence, and violet light (405 nm, excitation filter ET405/20x) was used to capture calcium-invariant hemodynamic occlusion. Excitation light was sent through the objective with a 3 mm core liquid light guide and dichroic mirror and emitted light was filtered (525/50–55) before the camera.

Widefield movies were compressed using singular value decomposition (SVD) of the form $F\;=\;USV^{T}$. The input to the SVD algorithm F was the *pixels x time* matrix of fluorescence values, and the outputs were U, the *pixels x components* matrix of spatial components, V, the *time x components* matrix of temporal components, and S, the diagonal matrix of singular values. The top 2000 components were retained, and all orthogonally invariant operations (such as deconvolution and averaging) were carried out on the matrix S∗V to save processing time and memory.

Hemodynamic effects on fluorescence were removed by subtracting a scaled violet illumination signal from the blue illumination signal. Fluorescence data was spatially downsampled 3-fold, filtered between 5-15 Hz to emphasize the heartbeat frequency, and sub-sample shifted to temporally align the alternating blue- and violet-illumination. A scaling factor was then regressed for each pixel from the violet to the blue illumination signal. The violet illumination signal was then multiplied by this scaling factor and subtracted from the blue illumination signal.

To correct for slow drift, hemodynamic-corrected fluorescence was then linearly detrended, high-pass filtered over 0.01 Hz, and ΔF/F_0_ normalized, where F_0_ was the average fluorescence by pixel across the session softened by the median fluorescence across pixels. Fluorescence was deconvolved using a kernel previously fit from simultaneous widefield imaging and electrophysiology (Peters et al., 2021).

To combine SVD-compressed widefield data across recordings, data was recast from experiment-specific SVD components into a master basis set of temporal components $U_{master}$, which was previously created from the spatial components of many mice. After aligning the spatial components within an experiment $U_{experiment}$ to the master alignment, temporal components ${\left(\text{S∗V}\right)}_{experiment}$ for each experiment were recast by

$${\left(S*V\right)}_{recast}=U_{master}^{T}*U_{experiment}*{\left(S*V\right)}_{experiment}$$

In this way, fluorescence data could be combined across experiments as temporal components V from a common basis set of spatial components $U_{master}$, greatly reducing processing time and memory.

#### Retinotopic mapping

Cortical visual areas were mapped using visual sparse noise stimuli. White squares 7.5° in length were presented asynchronously on a black background, with each square lasting 166 ms and ∼12% of squares being present at any given time. Activity for each square presentation was averaged within a 300–500 ms time window (corresponding to the maximum GcaMP6s signal), and the average response to each square was bootstrapped 10 times. Visual field sign maps (e.g. Figure S2A) were calculated for each bootstrapped mean and then averaged. Visual field sign was defined by gaussian-smoothing average square responses, finding the center-of-mass for each cortical pixel relative to the square azimuth and elevation locations, determining the gradient within these azimuth and elevation center-of-mass maps, and taking the sine of the difference between the azimuth and elevation gradients.

#### Widefield alignment

Widefield images were aligned across days within each mouse, and across mice to a master alignment, and to the Allen CCF atlas (Wang et al., 2020) (CCF v3, © 2015 Allen Institute for Brain Science) from the master alignment. Alignment across days was done using the average image within each day, first by finding vasculature edges through subtraction of a gaussian-blurred average image from the raw image, then by rigid-aligning these vasculature edges across days. Images were aligned across mice using retinotopic visual field sign maps. The visual field sign map for each mouse was affine aligned to a master visual field sign map previously created from an average and symmetrized map from many mice. The CCF atlas was aligned to the master visual field sign map by assigning expected visual sign values to each visual area on the atlas, then affine aligning the CCF visual sign map to the master visual sign map. Note the CCF alignment was only used to overlay area borders on widefield images and was not used for data processing.

#### Widefield movement-weighted hemisphere subtraction

We approximately isolated visually evoked fluorescence from movement-evoked fluorescence during task performance through a weighted hemisphere difference. Visually evoked activity is largely unilateral while movement evoked activity is largely bilateral (Mohajerani et al., 2010, Shimaoka et al., 2019) (Figure 2A), which allows us to subtract movement evoked activity in the following manner. We make two assumptions, first, that stimulus and movement are additive, which previous well-fitting linear models support (Coen et al., 2021; Peters et al., 2021; Shimaoka et al., 2019; Steinmetz et al., 2019), and second, that the timecourse of visually and movement-evoked activity are the same in the left and right hemisphere, which is evident in our data. From this, the fluorescence timecourse F(t) in a given region can be described as a sum of unilaterally specific visual (v) and motor (m) gains for bilaterally symmetric visual- (V(t)) or motor- (M(t)) related activity through F(t)=v∗V(t)+m∗M(t). The fluorescence for a given region in the left and right hemisphere then will be

$$F_{L}=v_{L}V+m_{L}M,\;F_{R}=v_{R}V+m_{R}M$$

As mice often make movements during the delay period while no visual stimuli are present, we can estimate the ratio between left and right movement components $m_{R}/m_{L}$. This ratiometric approach assumes that, while the total fluorescence for each movement may change depending on factors like movement vigor, the ratio in fluorescence between the left and right hemispheres remains consistent during counterclockwise wheel movements. Using this ratio, we can then estimate a signal proportional to only the visual signal, without the movement signal, in the left hemisphere

$$F_{L}=v_{L}V+m_{L}M,\;\frac{m_{L}}{m_{R}}F_{R}=\frac{m_{L}}{m_{R}}\left(v_{R}V+m_{R}M\right)$$

$$F_{L}-\frac{m_{L}}{m_{R}}F_{R}=v_{L}V+m_{L}M\;–\;\frac{m_{L}}{m_{R}}\left(v_{R}V+m_{R}M\right)$$

$$F_{L}-\frac{m_{L}}{m_{R}}F_{R}=v_{L}V+m_{L}M\;–\;\frac{m_{L}v_{R}}{m_{R}}V-\;\frac{m_{L}m_{R}}{m_{R}}M$$

$$F_{L}-\frac{m_{L}}{m_{R}}F_{R}=v_{L}V+m_{L}M-\frac{m_{L}v_{R}}{m_{R}}V-m_{L}M$$

$$F_{L}-\frac{m_{L}}{m_{R}}F_{R}=v_{L}V-\frac{m_{L}v_{R}}{m_{R}}V$$

$$F_{L}-\frac{m_{L}}{m_{R}}F_{R}=\left(v_{L}-\frac{m_{L}v_{R}}{m_{R}}\right)V$$

We determined the ratio $m_{R}/m_{L}$ using movements during delay periods that would have been rewarded had a stimulus been present, i.e. reaching the reward threshold for leftward movement without reaching the punish threshold for a rightward movement. For each mouse, we averaged the fluorescence for these delay period movements within each day, then averaged the resulting fluorescence across days, then fit a scaling factor between movement-evoked activity in the right and left hemisphere.

#### Electrophysiological recordings

Electrophysiological recordings were performed using Neuropixels 3A probes affixed to custom rods and moved with micromanipulators (Sensapex). Some mice were recorded across multiple days, with the same insertion point being targeted each day. For trained recordings, 5 previously trained and imaged mice were used, with one mouse being recorded for 4 days, two mice for 2 days, and 2 mice for 1 day. For naïve recordings, 4 naïve mice were habituated to the rig and stimulus for one day, then each mouse was recorded for 3 days.

To determine the probe trajectory, probes were coated in dye on the first day of recording by dipping in DiI (ThermoFisher) 5–6 times with a few seconds of air drying between dips. Data was collected using Open Ephys (Siegle et al., 2017), spike-sorted using Kilosort 2 (Stringer et al., 2019), and units representing noise were manually removed using Phy (Rossant et al., 2016).

Probe trajectories were reconstructed from histology by aligning histological slices to the Allen CCF atlas and manually tracing the dye track using publicly available custom code (https://github.com/petersaj/AP_histology). The endpoint of the probe cannot be reliably determined through histology and there were likely slight variations of probe depth across days, so the depth of the probe for each recording was determined with electrophysiological markers. During each recording, the top recording sites of the probe were deliberately left outside of the brain to provide a demarcation for the cortical surface. This demarcation was then identified in the data using the LFP signal, where the brain-external channels were highly correlated with each other and not correlated with brain-internal channels. The surface of the cortex was defined where there was a large drop in cross-channel LFP correlation starting at the top of the probe. This often corresponded to the location where the first units were detected. By applying this recording-specific depth to the histology-aligned trajectory, we could then determine the brain area for each probe site.

Multiunit activity (Figures 4B and 4C) was obtained by combining spikes from all units manually classified as non-noise within each area in each recording. For analysis and plotting, multiunit activity was normalized to difference in firing rate relative to baseline (ΔFR/FR_0_), where baseline firing rate FR_0_ was the average pre-stimulus firing rate in a given region softened by adding the 10^th^ percentile of baseline rates across all areas and recordings.

Single-unit analyses (Figures 4D–4F) were performed on a subset of high-quality units determined using publicly available custom code (https://github.com/Julie-Fabre/bombcell). High-quality units were defined with the following criteria:

1) Waveform has maximum of one trough and two peaks, with the trough preceding and being larger than the peak, which eliminates non-somatic units (Deligkaris et al., 2016)
2) Maximum waveform amplitude has a slope of > −20 across channels, which eliminates non-localized units
3) Waveform trough-to-peak duration is >100 μs and <800 μs, which eliminates noise units
4) Waveform amplitude during baseline is <30% the maximum amplitude, which eliminates noise units
5) Less than 20% of spikes are missing due to drift as estimated by a gaussian fit of spike amplitudes
6) At least 300 spikes are recorded
7) Less than 10% refractory period violations (false positives) estimated as *r* = 2^∗^(*τ*_R_ – *τ*_C_) ^∗^ *N*^2^^∗^ (1-*F*_p_) ^∗^ *F*_p_ / *T*, solving for *F*_p_, in which *τ*_R_ is the refractory period (set as 2 ms), *τ*_C_ is the censored period (set as 0.1 ms), *T* the total experiment duration, *r* the number of refractory period violations, *F*_p_ the fraction of contamination (Hill et al., 2011).

#### Behavioral camera analysis

Eye camera analysis was done using DeepLabCut (Mathis et al., 2018) (https://github.com/DeepLabCut/DeepLabCut) with an available model trained on pupil videos (https://github.com/sylviaschroeder/PupilDetection_DLC). Four markers were used to track the pupil, with pupil diameter being the average length between each pair of opposing markers. Only time points with at least two pupil markers over 80% likelihood were used, while other time points (e.g. when there was a blink) were excluded.

Whisker analysis was done by aligning images of mouse faces across all experiments by control point registration, then defining a linear region-of-interest across the whiskers. The pixels in the whisker region-of-interest were extracted for each video frame, and whisker movement was defined as the absolute value of the difference between consecutive frames, summed across pixels.

### Quantification and statistical analysis

#### Stimulus response statistics

To test whether the mouse was reacting to the stimulus, it was necessary to test whether the mouse had a shorter reaction time to the stimulus compared to chance. Reaction time here is defined as the time between the visual stimulus onset and the next wheel movement. The analysis of this question is complicated by the fact that, even if wheel turns occurred at random times, an increased rate of random wheel turns would lead to an apparent decrease in median reaction times. We therefore require a method that ascertains whether reaction times are faster than would be expected if wheel turns occurred at times unrelated to the visual stimuli, while accounting for changes in total turn rates.

To do this, we used a method based on conditional randomization. Conditional randomization is a very simple statistical framework, that surprisingly has seen little use until recently (Candès et al., 2018; Hennessy et al., 2016). This approach can be used when we do not know the full probability distribution of the observed data X, but can specify a null hypothesis for its conditional distribution given a conditioning statistic S(X). We compare the value of a test statistic T(X) to a null distribution obtained by randomly sampling of $T\left(X^{\prime }\right)$ from this conditional distribution $P\left[X^{\prime }|(S\left(X^{\prime }\right)=S\left(X\right))\right]$, rejecting the null hypothesis if the actual test statistic exceeds the 95^th^ percentile of this distribution.

In our case, the full data X consists of the full wheel movement timeseries for all trials, together with each trial’s delay parameters $D_{i}$: the lengths of the inter-trial interval and quiescent period, which are randomly chosen for each trial. Knowing this data, we can compute the time $V_{i}$ when the visual stimulus appeared on each trial i, and the time $M_{i}$ of the next movement occurring after the visual stimulus on this trial. We define the conditioning statistic S(X) to be the wheel movement timeseries for all trials, together with the observed movement times $M_{i}$. Thus, S(X) contains all information in X except for the delay parameters $D_{i}$. We can thus sample from the conditional distribution $P\left[X^{\prime }|(S\left(X^{\prime }\right)=S\left(X\right))\right]$ by rejection sampling: randomly redrawing the delay parameters for each trial, subject to the condition that the movement times that would have been detected with the new delay parameters are the same as those actually observed. For example, on a trial with a long delay between the stimulus onset $V_{i}$ and the next subsequent movement $M_{i}$, the same movement time would have been registered if the ITI was longer and the stimulus later; but on a trial with a short delay between $V_{i}$ and $M_{i}$, a longer ITI would have led to a stimulus coming later than $M_{i}$.

We created a null set of reaction times by 10,000-fold random resampling the delay parameters times for each trial, subject to the condition that the observed movement times $M_{i}$ would still have been registered. Our test statistic T(X) was the median across trials of the reaction times $M_{i}-V_{i}$, excluding reaction times less than 100 ms as these fast reaction times were likely the result of coincidental timing rather than being related to the stimulus. Reaction times were considered significantly faster than chance at p < 0.05. The first day that reaction times were significantly faster than chance for each mouse was considered the first “association day”.

#### Single-unit response classification

Single units were classified as being significantly responsive to stimuli or movements through a shuffle test comparing firing rate in a baseline window with a response window (Figure 4F). For both stimulus and movement onset, baseline windows were set at 500:300 ms before each event, and response windows were set at 0:200 ms after each stimulus onset and −100:100 ms around movement onset. Stimulus responses were obtained for all passive viewing trials with right-hand stimuli with no wheel movement. Movement responses were obtained from movements during task delay periods (with no stimulus present) that would have been rewarded had the stimulus been present (i.e., passing the counterclockwise threshold and not passing the clockwise threshold). The difference between baseline window and response window firing rate was compared to a null distribution, which was created by shuffling the baseline and response firing within each trial 1000 times, and cells were considered significantly responsive at p < 0.01.

## Data Availability

• Preprocessed data has been deposited at OSF and is publically available as of the date of publication. DOIs are listed in the key resources table. Other data reported in this paper will be shared upon request to the lead contact. • All original code has been deposited at Zenodo and is publicly available as of the date of publication. DOIs are listed in the key resources table. • Any additional information required to reanalyze the data reported in this paper is available from the lead contact upon request.

## Supplemental information

Document S1. Figures S1–S6

Document S2. Article plus supplemental information
