# Supplementary material for: Visuomotor learning promotes visually evoked activity in the medial prefrontal cortex
Source: Cell Rep. 2022 Oct 18;41(3):111487. doi: 10.1016/j.celrep.2022.111487 (PMC9631115; doi:10.1016/j.celrep.2022.111487)
Supplement: Document S1. Figures S1–S6 [file mmc1.pdf]

**Cell Reports, Volume *41***

## Supplemental information

# Visuomotor learning promotes visually evoked activity in the medial prefrontal cortex

**Andrew J. Peters, Andrada-Maria Marica, Julie M.J. Fabre, Kenneth D. Harris, and Matteo Carandini**

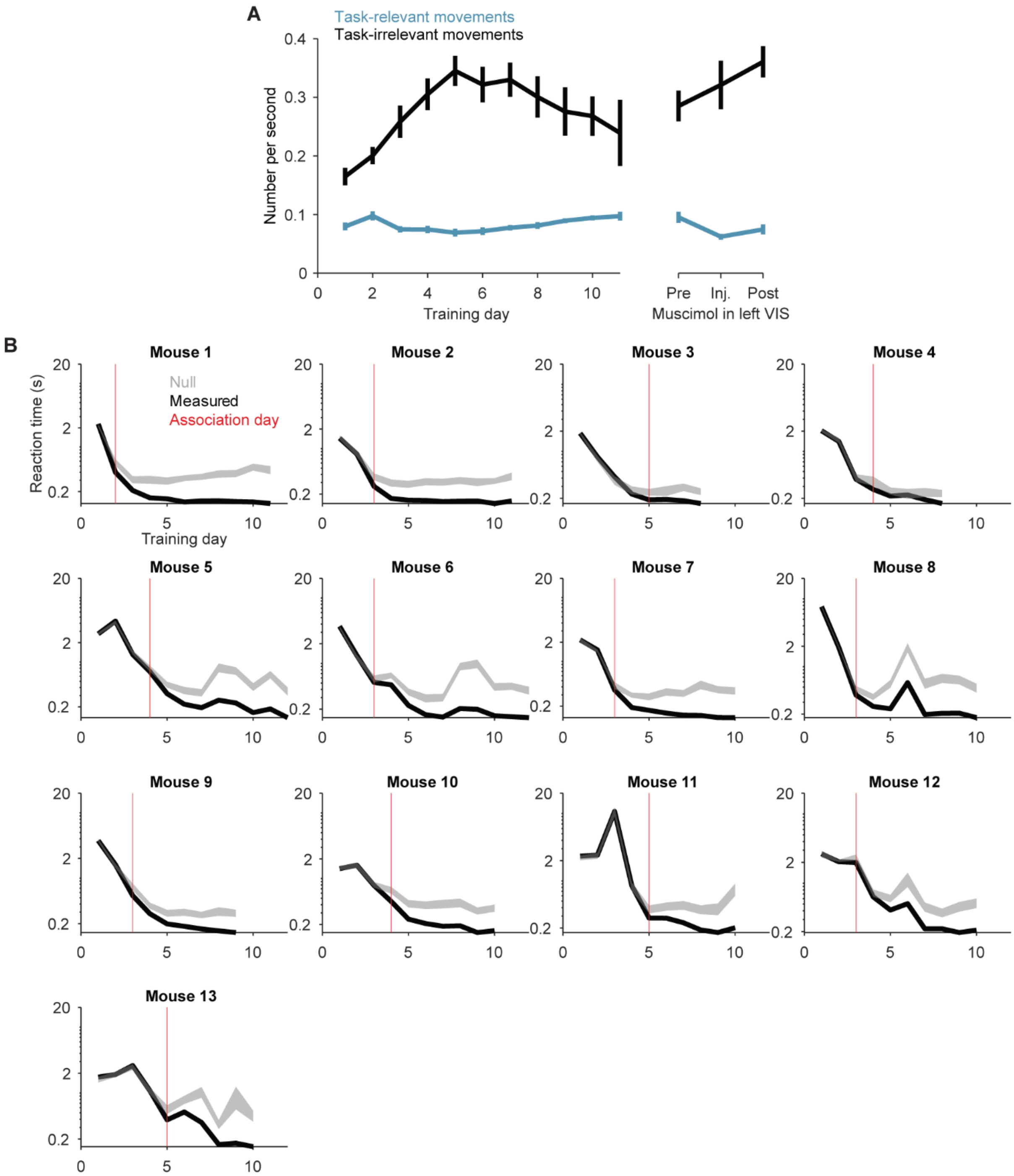


**Figure S1. Reaction times and movement rates. Related to Figure 1.**

(A) Left: rate of movements within each day, which were either task-relevant and triggered a reward or punishment (cyan), or were task-irrelevant and typically when there was no stimulus present (black). Curves and error bars show mean ± s.e. across mice (n = 13 mice). Mice increased the frequency of task-irrelevant movements in the first week (one-way ANOVA, $p = 1.60 \times 10^{-4}$). Right: movement rates for days before (pre), on (inj.), and after (post) muscimol injection into the left primary visual cortex. Inactivating the visual cortex did not change task-irrelevant movement rate (one-way ANOVA, p = 0.29) and slightly reduced task-relevant movement rate (one-way ANOVA, p = 0.03).

(B) As in Figure 1F, median reaction times across days, measured (black) or expected from chance (gray) separately for all mice (n = 13 mice). Curves are measured values; shading shows 95% confidence intervals from the null distribution that accounts for changes in overall movement rates. Reaction times too fast to be stimulus-responsive (< 100ms) are excluded. Red lines indicate the first day that reaction times diverged from the null which represent the "association day", which were used to split days by group in Figure 2 and to align by association day in Figure 3.

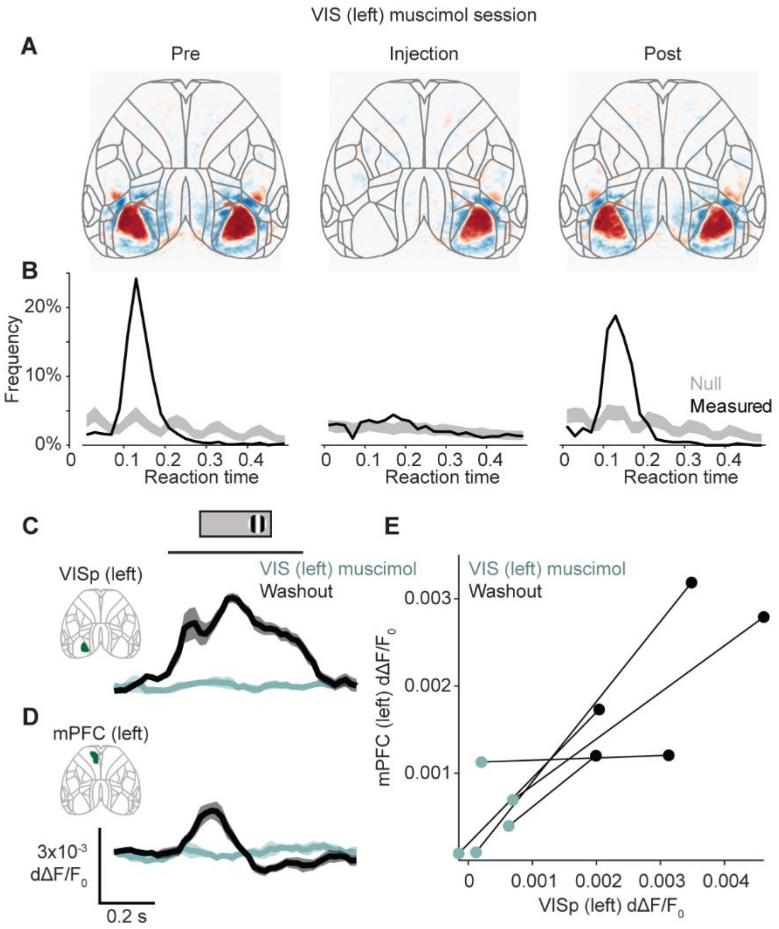


**Figure S2. Muscimol abolishes visual and mPFC responses and movement timing without affecting total movement. Related to Figures 1 and 2.**

(A) Visual field sign maps obtained from sparse noise visual presentation on days before (left), on (center), and after (right) muscimol injection in the left visual cortex averaged across mice (n = 5 mice). Muscimol injections into the left visual cortex abolishes retinotopic visual responses.

(B) Histograms of reaction times measured (black) or expected from chance (null) as in Figure 1D. Curves are mean across mice and shadings are 95% confidence intervals from the null distribution (n = 5 mice).

(C) Fluorescence in the left visual cortex during passive viewing of right-hand stimuli, on (cyan) and after (black) days with muscimol injection into the left visual cortex. Curves and error bars show mean ± s.e.m. across mice (n = 5 mice).

(D) As in (C), for mPFC.

(E) Fluorescence in the left visual cortex and mPFC as the maximum within 0-200 ms after stimulus onset, each connected pair is one mouse (n = 5 mice). Inactivating the visual cortex reduces stimulus-evoked responses in the mPFC (left-sided signed-rank test, $p = 0.031$).

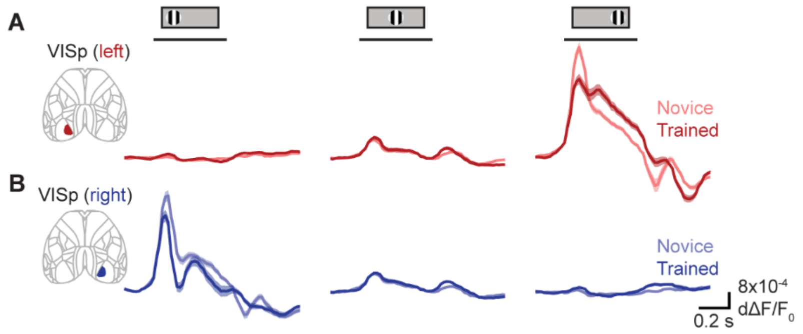


**Figure S3. Left VISp fluorescence to right-hand stimulus becomes sustained with training. Related to Figure 2.**

(A) Fluorescence during passive stimulus viewing in left hemisphere primary visual cortex (VISp) in novice (light red) and trained (dark red) mice. Curves and shading show mean ± s.e.m. across mice. Line under stimulus icon indicates when the stimulus is on the screen. Fluorescence in the left VISp to contralateral (right-hand) stimuli decreases in onset amplitude but becomes more sustained with learning (three-way ANOVA on time, learning stage, and stimulus, learning stage effect $p = 1.2 \times 10^{-3}$).

(B) As in (A), for the right hemisphere VISp. Fluorescence in the right VISp to contralateral (left-hand) stimuli reduces in onset amplitude with learning (three-way ANOVA on time, learning stage, and stimulus, learning stage effect $p = 0.013$).

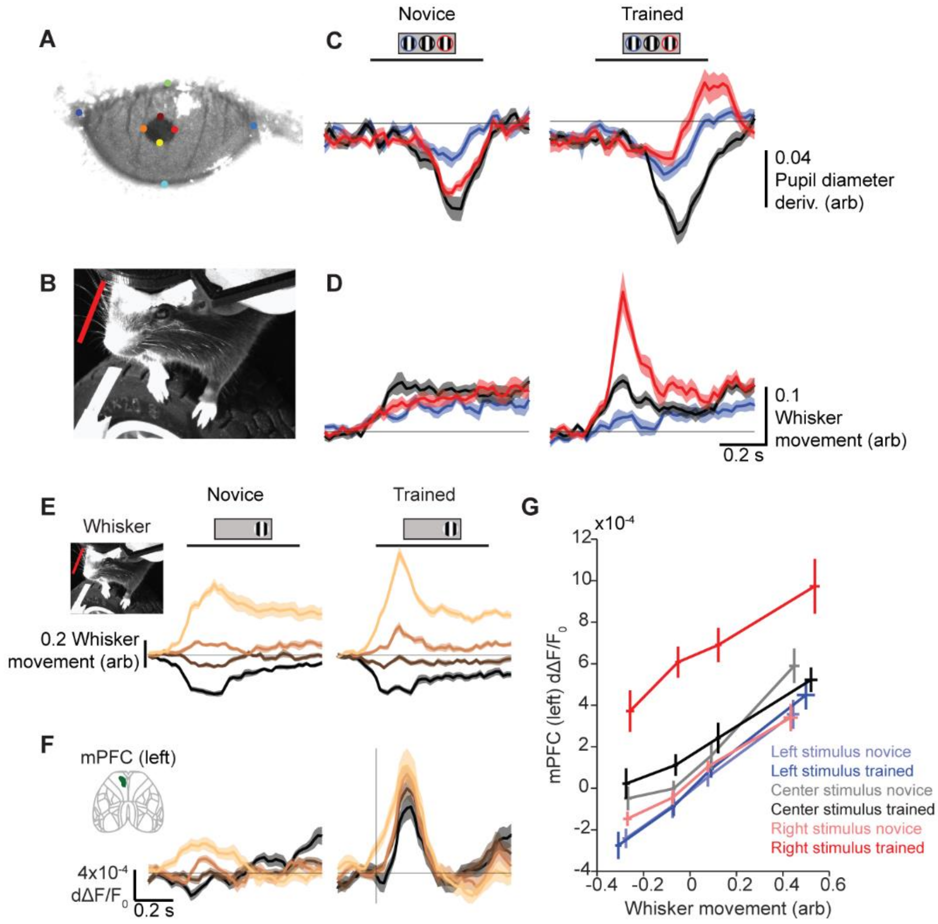


**Figure S4. mPFC stimulus-evoked responses after learning are robust to behavioral changes. Related to Figure 2.**

(A) Example eye video with points marked from DeepLabCut annotation.

(B) Example face video with whisker region-of-interest in red.

(C) Change in pupil diameter during passive viewing of stimuli on left (blue), center (black), and right (red) during novice (left) and trained (right) days. Curves are average ± s.e.m. across mice (n = 13).

(D) As in (C), for whisker movement.

(E) Whisker movement aligned to stimulus onset during novice (top) and trained (bottom) stages, binned into quartiles for each session by maximum movement 0-200 ms after stimulus onset.

(F) Fluorescence in the left hemisphere mPFC from trials binned by whisker movement in (E).

(G) Fluorescence in left mPFC by whisker movement, each averaged 0-200 ms after stimulus onset and binned by whisker movement in (E). Colors are stimuli on the left (blue), center (black), and right (red), for novice (pale) and trained (dark) stages. Curves are average ± s.e.m. across mice. Fluorescence increases in left mPFC specifically to right-hand stimuli regardless of movement (two-way ANOVA, stage effect left-hand stimulus $p = 0.55$, center stimulus $p = 0.27$, right-hand stimulus $p = 1.3 \times 10^{-18}$).

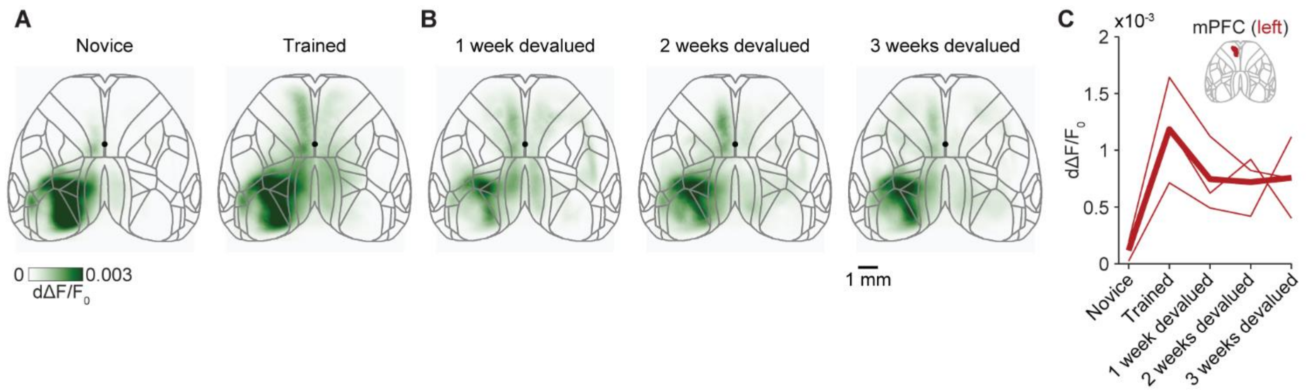


**Figure S5. mPFC stimulus-evoked responses persist after devaluation. Related to Figure 2.**

(A) Maximum fluorescence 0-200 ms after stimulus onset in passive condition, averaged across mice (n = 3) in the novice and trained learning stage (as in Figure 2E).

(B) As in (A), 1, 2, and 3 weeks after ending training sessions and providing the mice with *ad lib* water access, thereby devaluing the stimulus as mice were no longer thirsty.

(C) Fluorescence in the left mPFC to passive presentation of right-hand stimuli from the time points in (A-B). Increased passive stimulus responses in the mPFC do not change significantly through long-term devaluation (one-way ANOVA of timepoints from the trained stage through devaluation, $p = 0.39$), and responses after devaluation were larger than in the novice stage (rank-sum test, $p = 9.1 \times 10^{-3}$)). Thin lines are individual mice, thick line is average across mice.

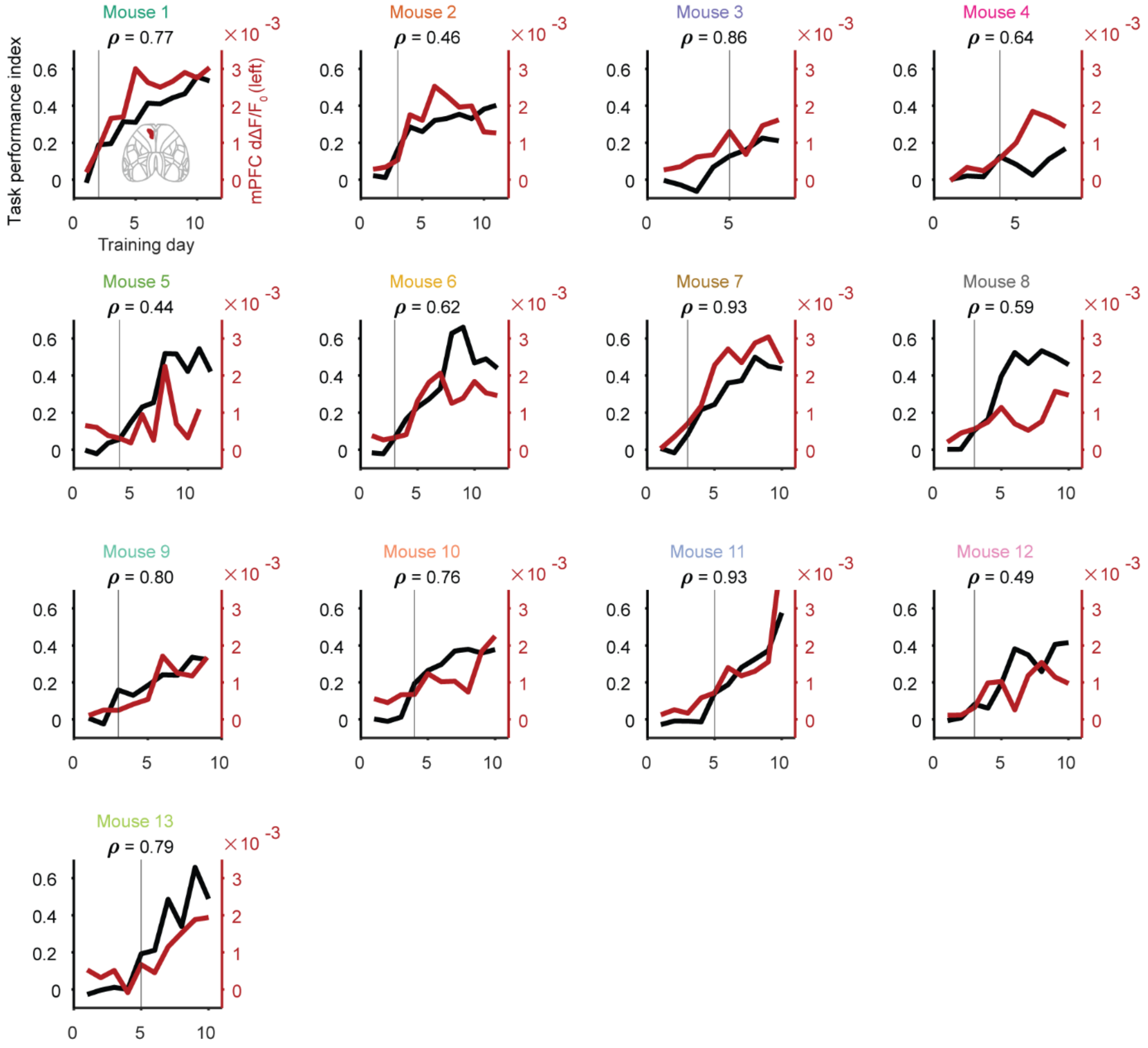


**Figure S6. History of sensorimotor association correlates with history of passive mPFC stimulus-evoked activity for each mouse. Related to Figure 3.**

Data from Figure 3F, separately for each mouse. Task performance index during the task (black lines) and fluorescence in the left mPFC to passive viewing of right-hand stimuli immediately after training on each day (red lines), vertical lines are association days. Task performance index is defined as the difference divided by the sum of actual and chance median reaction times, where positive reaction time index indicates shorter reaction times than chance. $\rho$ is Spearman's correlation coefficient between reaction time index and mPFC fluorescence within each mouse.
